# Ideal cardiovascular health metrics have better identification of arthritis

**DOI:** 10.1186/s12889-023-17602-7

**Published:** 2024-01-08

**Authors:** Yuxiang Wang, Mengzi Sun, Nan Yao, Shifang Qu, Ruirui Guo, Xuhan Wang, Jing Li, Zechun Xie, Yan Liu, Zibo Wu, Fengdan Wang, Bo Li

**Affiliations:** Department of Epidemiology and Biostatistics, School of Public Health, Jilin University, 1163 Xinmin Avenue, 130021 Changchun, P. R. China

**Keywords:** Arthritis, Different types of arthritis, Forest plot, Ideal Cardiovascular Health Metrics, Mixed graphical model

## Abstract

**Background:**

This study aimed to explore the association between ideal cardiovascular health metrics (ICVHM) and arthritis (AR), as well as the interactions of various indicators in ICVHM on AR in US adults.

**Methods:**

We involved 17,041 participants who were interviewed by NHANES from 2011 to 2018. AR included osteoarthritis or degenerative arthritis (OA), rheumatoid arthritis (RA), and psoriatic arthritis and other arthritis (Other AR). Logistic regression was applied to analyze the association between AR and ICVHM. Mixed graphical model (MGM) was used to explore the interaction between variables in ICVHM.

**Results:**

Higher ICVHM scores had a protective effect on AR. Compared to “≤1” score, the ORs of AR in participants with 2, 3, 4, and ≥5 were 0.586, 0.472, 0.259, and 0.130, respectively. Similar results were also found in different types of AR. ICVHM has a maximum area under the curve value of 0.765 and the interaction between blood pressure and total cholesterol was 0.43.

**Conclusions:**

ICVHM correlates significantly with AR and is better at identifying AR than individual indicators. ICVHM can be better improved by controlling the indicators with stronger interactions. Our findings provide guidance for promoting health factors, which have important implications for identification and prevention of AR.

**Supplementary Information:**

The online version contains supplementary material available at 10.1186/s12889-023-17602-7.

## Introduction

Arthritis (AR) means inflammation or swelling of one or more joints [1], mainly including osteoarthritis (OA) and rheumatoid arthritis (RA). AR could cause pain and limited mobility in the joints [1, 2], as well as extra-articular manifestations such as rheumatoid nodules, other systemic involvement, and systemic comorbidities [3]. An estimated 58.5 million U.S. adults (22.7%) were told by their doctors each year from 2013 to 2015 that they had some form of AR [4]. According to relevant studies, the prevalence of AR and the limitation of activities caused by it will increase significantly by 2030 [5]. As a major public health problem, AR had brought a heavy medical and economic burden.

The American Heart Association (AHA) proposed the concept of ICVHM, which was based on seven health metrics [6]. Previous studies had confirmed the association between ICVHM and cardiovascular disease (CVD) [7, 8]. AR and CVD share risk factors as well as biological and behavioral mechanisms [9–11].

Previous studies have pointed out that some indicators in ICVHM were associated with AR. Senbo Zhu et al [12] and Milena A. Gianfrancesco et al [13] studied the relationship between smoking with OA and RA, respectively. A review confirms that arthritis-induced pain can be relieved by physical activity [14]. Sally Thomas et al. provide evidence of a role in osteoarthritis through diet [15]. Karen Ching et al [16] suggested a strong association between hypertension and arthritis and raised the possibility of using anti-hypertensive drugs to treat osteoarthritis. However, there is still no comprehensive index to clarify the association of these single indicators with AR. Fortunately, ICVHM is a comprehensive indicator that includes multiple factors (smoking, diet, physical activity, BMI, blood pressure, blood glucose, and total cholesterol) [6]. Thus, we used ICVHM to investigate their association and the possibility of identifying AR. We hypothesized that there is an association between ICVHM and AR, and that ICVHM may better identify AR. Therefore, this study aimed to explore the relationship between AR (and its different types) and ICVHM, as well as the interactions of various indicators in ICVHM on AR in US adults.

## Materials and methods

### Study population

The study sample consists of four continuous cycles (2011– 2012, 2013–2014, 2015–2016, and 2017–2018) of NHANES, which were conducted by the Centers for Disease Control and Prevention (CDC) to assess the health of non-institutional residents of the United States [17]. A total of 39,156 individuals participated in NHANES during 2011–2018. After the exclusion of 16,590 participants with missing data on AR, 3620 participants with missing data on ICVHM, 1704 participants with missing data on covariates, and 201 pregnant participants, a total of 17,041 participants were eventually involved in the study, the data filtering flow chart was shown in Supplementary Figure S1. The protocols of NHANES were approved by the institutional review board of the National Center for Health Statistics. All the participants signed the informed consent before participating in the study.

### Assessment of ICVHM

According to the recommendations of the Goals and Metrics Committee of the Strategic Planning Task Force of the AHA [6], ICVHM was defined as consisting ideal status of four lifestyle factors (i.e., smoking, physical activity, diet, and BMI) and three additional cardiometabolic factors (i.e., blood pressure, blood glucose, and total cholesterol). Therefore, participants who met the above criteria were classified as the healthy group, and who did not meet the requirements were classified as the unhealthy group. The healthy group was assigned a value of 1 and the unhealthy group was assigned a value of 0, the ICVHM score was the sum of the individual indicators, ranging from 0 to 7. We defined participants who smoke less than 100 cigarettes in their lifetime as a healthy group [18]. Meanwhile, according to the questionnaire design, participants with moderate or vigorous physical activity in a week were identified as the healthy group. Dietary quality data from the NHANES database, obtained by 24-hour dietary recall, were assessed by the Healthy Eating Index (HEI) score, which reflected overall diet quality, with distribution in the top two quintiles considered healthy [18–20]. In addition, according to the ICVHM definition, participants with BMI < 25.0 kg/m^2^ were classified as the healthy group [6].

We calculated the average of three measurements of blood pressure, ideal blood pressure was defined as self-reported no hypertension or prehypertension and blood pressure < 120/80 mmHg [21]. Ideal blood glucose was defined as no reported hyperglycemia or pre-hyperglycemia, and fasting blood glucose < 100 mg/dl [22]. Total cholesterol was defined as ideal if self-reported no high cholesterol level and total cholesterol < 200 mg/dl [23] and details were described in Supplementary Table S1.

### Mixed graphical model (MGM)

The MGM is mainly applicable to complex relationships between multiple variables and interactions between different variables. We applied the ‘mgm’ package to the network estimation of the MGM [24]. The data are visualized to describe the strength and direction of the correlation [25]. The parameters between two categorical variables correspond to the interactions between two corresponding indicator variables. Nodes represent variables and edges reflect their interactions [24].

### Assessment of arthritis

AR status was determined by the answers to the questionnaire on medical conditions. In this part, participants were asked if s/he had been ever told by a doctor or health professional that s/he had AR and which type of AR was it. Participants who answered that a doctor or health professional had told s/he had arthritis was defined as “AR”. Participants who self-reported “osteoarthritis or degenerative arthritis” was defined as “OA”, “rheumatoid arthritis” as “RA”, and “psoriatic arthritis and other arthritis” as “Other AR”.

### Statistical analysis

Data management and statistical analysis were performed using SPSS 24.0 and plotting using R 4.2.0 and Cytoscape3.7.1. All analyses were complexly weighted to ensure their representativeness. We use frequency and percentage to characterize cross-sectional subjects. The classification variables were compared by the chi-square test. Logistic regression analysis was used to calculate the odds ratio (OR) and 95% confidence interval (CI) to analyze the association between ICVHM and AR and its different types. The MGM was used to describe the interactions between the variables in ICVHM. All statistical tests were two-sided test, *P* values less than 0.05 were considered statistically significant.

## Results

Table 1 showed the background characteristics and the prevalence of AR in adult respondents. Among 17,041 participants, 4566 (26.4%) had AR. In addition, participants with ICVHM scores ≤ 1, 2, 3, 4, and ≥ 5 accounted for 15.2%, 19.2%, 23.0%, 19.0%, and 23.6%, respectively. The distribution of age, gender, race, and education was significantly different between AR patients and non-AR participants, as well as between different ICVHM score groups (*P* < 0.001).

**Table 1:** Background characteristics and the prevalence of AR in adult respondents

Variables		n (weighted%)	AR		χ^2^	*P*
			yes[n (weighted%)]	no[n (weighted%)]		
**AR**	Yes	4566 (26.4)				
	No	12475 (73.6)				
**Age**	≤ 65	13468 (82.8)	2667 (16.7)	10801 (66.0)	1627.630	< 0.001
	> 65	3573 (17.2)	1899 (9.7)	1674 (7.5)		
**Gender**	males	8385 (49.0)	1845 (10.5)	6540 (38.5)	208.644	< 0.001
	females	8656 (51.0)	2721 (15.9)	5935 (35.1)		
**Race**	non-Hispanic White	6776 (66.2)	2292 (20.0)	4484 (46.1)	252.001	< 0.001
	other race	10265 (33.8)	2274 (6.4)	7991 (27.4)		
**Education**	high school or below	12655 (68.6)	3668 (19.6)	8987 (49.0)	82.863	< 0.001
	college or above	4386 (31.4)	898 (6.9)	3488 (24.5)		
**Marital status**	married	10085 (62.0)	2536 (16.5)	7549 (45.5)	0.428	0.733
	other	6956 (38.0)	2030 (9.9)	4926 (28.1)		
**PIR**	< 2.5	9615 (44.1)	2732 (11.9)	6883 (32.1)	2.407	0.409
	≥ 2.5	7426 (55.9)	1834 (14.5)	5592 (41.4)		
**Drinking**	yes	2473 (16.8)	551 (4.1)	1922 (12.7)	8.333	0.084
	no	14566 (83.2)	4015 (22.4)	10553 (60.8)		
**ICVHM scores**	≤ 1	2871 (15.2)	1369 (7.3)	1502 (7.9)	1478.370	< 0.001
	2	3488 (19.2)	1240 (6.8)	2248 (12.4)		
	3	3876 (23.0)	1069 (6.7)	2807 (16.3)		
	4	3179 (19.0)	561 (3.4)	2618 (15.6)		
	≥ 5	3627 (23.6)	327 (2.3)	3300 (21.4)		

**Fig. 1:**
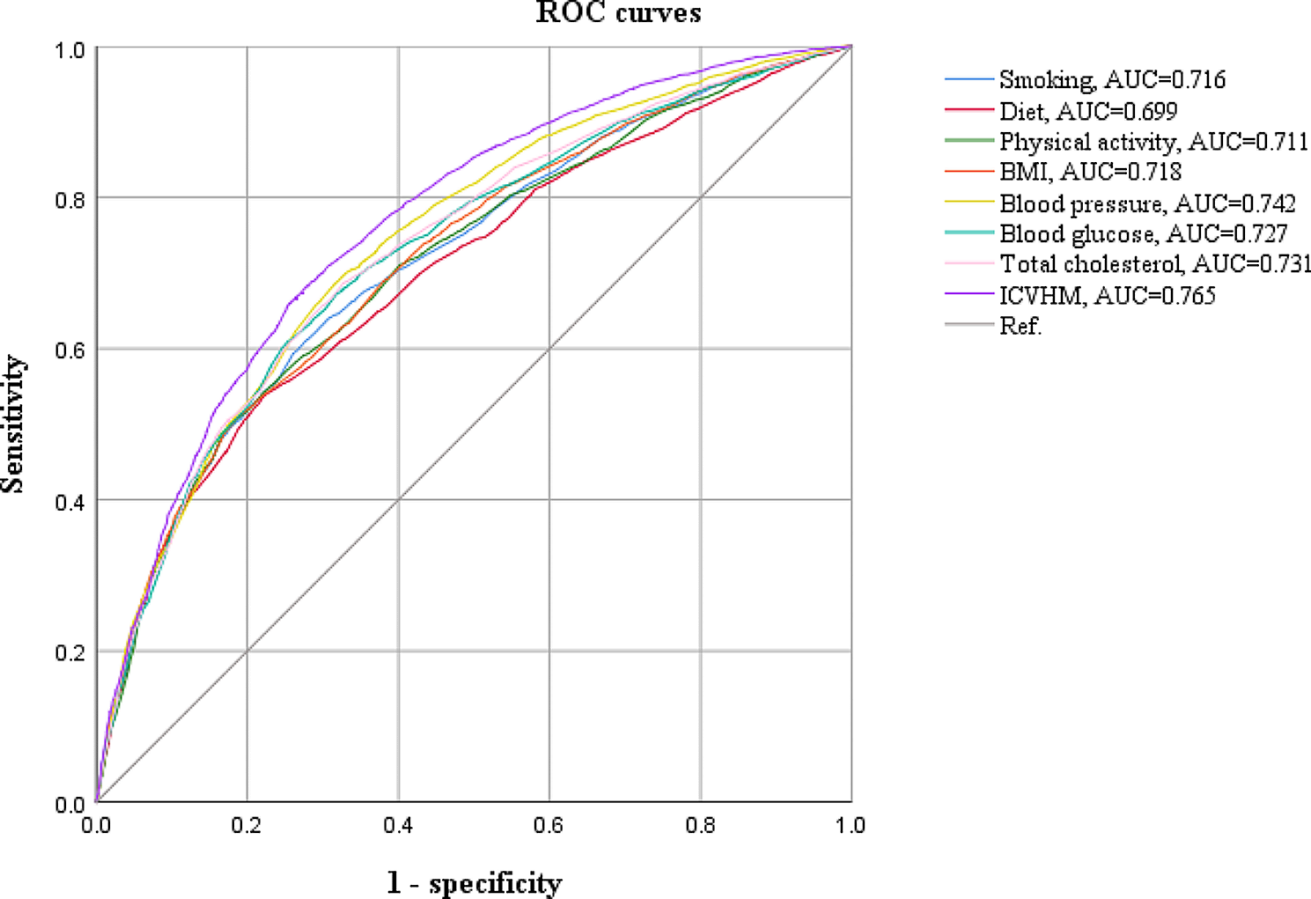
Area under the curve values for ICVHM and its parameters in the identification of AR

We used multivariate logistic regression analysis to explore the effects of seven independent health factors and ICVHM scores, and the results were shown in Table 2. Compared to the unhealthy groups, the ORs in the health status of smoking, physical activity, BMI, blood pressure, blood glucose, and total cholesterol were 0.598, 0.603, 0.479, 0.348, 0.441, and 0.491, respectively. Figure 1 shows the area under the curve (AUC) values for ICVHM and its parameters in the identification of AR. ICVHM as a composite has a maximum AUC value of 0.765, which is higher than the AUC value of the individual indicators.

**Table 2:** Odds ratio (OR) and 95% confidence interval (CI) for AR according to each ICVHM

Metrics		Model 1^a^	Model 2^b^	Model 3^c^
**Smoking**	unhealthy	1.000	1.000	1.000
	healthy	0.586 (0.530,0.647)	0.604 (0.538,0.679)	0.598 (0.532,0.672)
**Diet**	unhealthy	1.000	1.000	1.000
	healthy	1.013 (0.905,1.135)	0.916 (0.815,1.029)	0.916 (0.814,1.032)
**Physical activity**	unhealthy	1.000	1.000	1.000
	healthy	0.535 (0.472,0.607)	0.603 (0.530,0.686)	0.603 (0.529,0.687)
**BMI**	unhealthy	1.000	1.000	1.000
	healthy	0.496 (0.434,0.568)	0.480 (0.420,0.548)	0.479 (0.419,0.548)
**Blood pressure**	unhealthy	1.000	1.000	1.000
	healthy	0.290 (0.255,0.329)	0.349 (0.302,0.403)	0.348 (0.301,0.402)
**Blood glucose**	unhealthy	1.000	1.000	1.000
	healthy	0.350 (0.305,0.402)	0.442 (0.381,0.511)	0.441 (0.381,0.511)
**Total cholesterol**	unhealthy	1.000	1.000	1.000
	healthy	0.438 (0.399,0.482)	0.492 (0.444,0.544)	0.491 (0.443,0.544)
**ICVHM score**	≤ 1	1.000	1.000	1.000
	2	0.586 (0.494,0.695)	0.586 (0.483,0.711)	0.586 (0.483,0.710)
	3	0.445 (0.387,0.513)	0.472 (0.403,0.552)	0.472 (0.403,0.552)
	4	0.236 (0.198,0.281)	0.259 (0.214,0.313)	0.259 (0.214,0.313)
	≥ 5	0.114 (0.093,0.140)	0.130 (0.103,0.164)	0.130 (0.103,0.164)

^a^ Model 1 The covariates were not adjusted.

^b^ Model 2 Adjusted the covariates of age, gender, race, education, marital status and PIR.

^c^ Model 3 Adjusted the covariates of age, gender, race, education, marital status, PIR and drinking.

And compared to participants who “≤ 1” score, the ORs of AR in participants with 2, 3, 4, and ≥ 5 for AR according to ICVHM scores were 0.586, 0.472, 0.259, and 0.130, respectively. In subgroup analysis, compared with participants with ≤ 1 score, the ORs of AR in the age ≤ 65 and > 65 years old were 0.097 and 0.434 who met ≥ 5 score. In the male and female groups, compared with participants with ≤ 1 score, the ORs of participants with ≥ 5 score were 0.208, and 0.100, respectively. Similar results were observed in subgroups stratified by race, marital status, education, ratio of family income to poverty (PIR), and drinking (Table 3). In addition, we also found ICVHM had interaction effects between age, gender, race, and ICVHM on AR (*P* < 0.05). In the age ≤ 65 years old group, the protective effect of ICVHM on AR was relatively stronger than in the elderly. Similarly, the OR was generally smaller in females than that in males. Compared to non-Hispanic whites, ICVHM had a stronger protective effect on AR in other races.

**Table 3:** Subgroup analysis of multivariable-adjusted ORs (95% CIs) for AR by ideal cardiovascular health metrics

Subgroups	ICVHM scores, OR (95%CI)					*P* ^a^	*P* ^b^
	≤ 1	2	3	4	≥ 5		
**Age**							< 0.001
≤ 65	1.000	0.529 (0.430,0.652)	0.419 (0.341,0.516)	0.204 (0.160,0.259)	0.097 (0.073,0.130)	< 0.001	
> 65	1.000	0.765 (0.574,1.018)	0.635 (0.477,0.846)	0.529 (0.363,0.771)	0.434 (0.281,0.669)	< 0.001	
**Gender**							0.011
males	1.000	0.563 (0.440,0.720)	0.509 (0.407,0.636)	0.301 (0.226,0.401)	0.208 (0.147,0.294)	< 0.001	
females	1.000	0.590 (0.447,0.779)	0.428 (0.337,0.542)	0.228 (0.176,0.297)	0.100 (0.073,0.137)	< 0.001	
**Race**							0.041
non-Hispanic White	1.000	0.612 (0.480,0.780)	0.524 (0.428,0.640)	0.275 (0.217,0.347)	0.150 (0.114,0.196)	< 0.001	
other race	1.000	0.521 (0.428,0.636)	0.363 (0.277,0.474)	0.224 (0.172,0.291)	0.087 (0.066,0.115)	< 0.001	
**Education**							0.440
high school or below	1.000	0.569 (0.470,0.689)	0.436 (0.358,0.530)	0.234 (0.190,0.288)	0.117 (0.088,0.155)	< 0.001	
college or above	1.000	0.704 (0.455,1.089)	0.658 (0.447,0.970)	0.365 (0.237,0.563)	0.185 (0.113,0.303)	< 0.001	
**Marital status**							0.061
married	1.000	0.509 (0.401,0.646)	0.416 (0.334,0.517)	0.235 (0.179,0.309)	0.133 (0.098,0.181)	< 0.001	
other	1.000	0.744 (0.569,0.973)	0.590 (0.465,0.750)	0.321 (0.238,0.433)	0.128 (0.089,0.185)	< 0.001	
**PIR**							0.400
< 2.5	1.000	0.617 (0.491,0.774)	0.447 (0.364,0.548)	0.223 (0.181,0.275)	0.116 (0.084,0.162)	< 0.001	
≥ 2.5	1.000	0.566 (0.408,0.784)	0.499 (0.380,0.655)	0.292 (0.212,0.401)	0.142 (0.097,0.206)	< 0.001	
**Drinking**							0.163
yes	1.000	0.736 (0.461,1.175)	0.825 (0.512,1.329)	0.364 (0.202,0.656)	0.160 (0.092,0.278)	< 0.001	
no	1.000	0.568 (0.464,0.696)	0.426 (0.358,0.507)	0.245 (0.199,0.303)	0.126 (0.098,0.162)	< 0.001	

^a^*P* value for trend.

^b^*P* value for interaction.

Trend test for ICVHM scores were performed by treating the score of ideal cardiovascular health metrics as a continuous variable.

We further analyzed the relationship between ICVHM scores and different types of AR and made the results more intuitive by plotting the forest plot (Figure S2). Whether it was OA, RA, or Other AR, the *P*-trend of ICVHM scores was < 0.001, and compared with ICVHM score ≤ 1, the ORs of ≥ 5 were 0.174, 0.224, and 0.188, respectively.

Our study also explored the association between ICVHM and different types of AR in different characteristic populations, the results of the logistic analysis were shown in Table 4. In the age > 65 years old, the OR of the association between OA and ICVHM was 0.874, slightly higher than 0.649 in the age ≤ 65 years old. And in different genders, their associations were still significant (all *P* values were < 0.001). The association between RA and ICVHM was significant in the age ≤ 65 years old (*P* < 0.001), but not in the age > 65 years old (*P* = 0.956). And the ORs in the males and females were 0.770 and 0.730, respectively. For Other AR, in the males and females, the ORs were 0.733 and 0.689, as well as in the age ≤ 65 and > 65 years old, the ORs were 0.699 and 0.726, respectively. There were interaction effects between ICVHM and age for both OA and RA, and only interaction between race and ICVHM on OA.

**Table 4:** Subgroup analysis of multivariable-adjusted ORs (95% CIs) for each type of AR by ideal cardiovascular health metrics

Subgroups	OA			RA			Other AR		
	OR (95%CI)	*P* ^a^	*P* ^b^	OR (95%CI)	*P* ^a^	*P* ^b^	OR (95%CI)	*P* ^a^	*P* ^b^
**Age**			< 0.001			< 0.001			0.394
≤ 65	0.649 (0.602,0.701)	< 0.001		0.689 (0.637,0.744)	< 0.001		0.699 (0.623,0.785)	< 0.001	
> 65	0.874 (0.799,0.955)	0.004		1.004 (0.870,1.158)	0.956		0.726 (0.588,0.897)	0.001	
**Gender**			0.050			0.937			0.647
males	0.759 (0.689,0.836)	< 0.001		0.770 (0.667,0.889)	< 0.001		0.733 (0.619,0.867)	< 0.001	
females	0.676 (0.636,0.718)	< 0.001		0.730 (0.663,0.803)	< 0.001		0.689 (0.611,0.778)	< 0.001	
**Race**			0.011			0.138			0.491
non-Hispanic White	0.730 (0.686,0.776)	< 0.001		0.790 (0.716,0.872)	< 0.001		0.722 (0.635,0.822)	< 0.001	
other race	0.605 (0.557,0.658)	< 0.001		0.662 (0.614,0.715)	< 0.001		0.658 (0.594,0.729)	< 0.001	
**Education**			0.054			0.193			0.494
high school or below	0.667 (0.620,0.718)	< 0.001		0.753 (0.696,0.814)	< 0.001		0.711 (0.627,0.807)	< 0.001	
college or above	0.782 (0.716,0.853)	< 0.001		0.691 (0.554,0.863)	0.001		0.668 (0.54,0.826)	< 0.001	
**Marital status**			0.185			0.516			0.852
married	0.708 (0.656,0.764)	< 0.001		0.706 (0.632,0.787)	< 0.001		0.719 (0.621,0.833)	< 0.001	
other	0.700 (0.653,0.750)	< 0.001		0.803 (0.722,0.894)	< 0.001		0.688 (0.592,0.800)	< 0.001	
**PIR**			0.800			0.716			0.835
< 2.5	0.686 (0.645,0.731)	< 0.001		0.730 (0.656,0.813)	< 0.001		0.677 (0.589,0.778)	< 0.001	
≥ 2.5	0.716 (0.659,0.779)	< 0.001		0.766 (0.673,0.871)	< 0.001		0.732 (0.638,0.840)	< 0.001	
**Drinking**			0.502			0.287			0.520
yes	0.725 (0.650,0.808)	< 0.001		0.847 (0.688,1.041)	0.113		0.597 (0.453,0.787)	< 0.001	
no	0.698 (0.656,0.742)	< 0.001		0.726 (0.669,0.786)	< 0.001		0.727 (0.648,0.816)	< 0.001	

^a^*P* value for trend.

^b^*P* value for interaction.

Trend test for ICVHM scores were performed by treating the score of ideal cardiovascular health metrics as a continuous variable.

The interaction diagram between variables is shown in Figure S3 and Table S2 in the supplement. We observed that there is an interaction between each indicator, and the strongest interaction is blood pressure and total cholesterol (0.43), followed by blood pressure and blood glucose (0.34), BMI and blood pressure (0.32), etc.

## Discussion

In this cross-sectional study, which included 17,041 U.S. adults, we found a high prevalence of AR of 26.8% and a negative association between ICVHM scores and the prevalence of AR, including OA, RA and other AR. ICVHM has the highest AUC value when compared to a single indicator. There were interactions between age, gender, race, and ICVHM on AR. These observations suggest an association between ICVHM and AR, with better identification of AR compared to a single indicator.

In this study, we found that smoking, physical activity, BMI, blood pressure, blood glucose, and total cholesterol were independently and negatively associated with AR among the single variables constituting ICVHM. These indicators may influence the development and progression of arthritis through potential mechanisms. Similar to previous studies, the study of Sugiyama et al [26], smoking had been reported to be associated with an increased risk of RA, which may be related to the formation of citrullination of antigens and the formation of anti-citrullinated peptide antibodies (ACPAs) in RA [27, 28]. There was clear evidence that physical activity was associated with a variety of diseases, and physical activity had also been recommended for a healthy population and AR patients [29, 30]. In our research, the same results have been obtained. In a Swiss study, obese participants had higher rates of AR and significantly more pain than non-obese participants, which may be related to less physical activity in the obese population [31]. There was a strong association between high blood pressure and AR, and it was also common comorbidity of AR [16, 32, 33], which may be related to higher concentrations of homocysteine and leptin [34], but the specific mechanism needs to be further studied. Our study also confirmed the association between blood glucose and AR, and the meta-analysis by Karin et al [35]and Hui Pi et al [36], highlighted the association of glucose metabolism with OA and RA. However, in the study of Lusen et al [37], the opposite view was proposed, suggesting that the association between AR and blood glucose may be due to the presence of confounding factors such as obesity. Similarly, our findings support the association between total cholesterol and AR [38]. In this study, no independent association between diet and AR was found. However, different from our results, a clinical trial proved that diet was better than exercise alone in relieving AR symptoms [39], Sajedeh Jandari et al [40] demonstrated the association of the dietary inflammatory index(DII) and HEI with RA in their study, which may be related to the inconsistent relationship between AR and different nutrients in the diet [41], and the inconsistent grouping of the healthy diet. Meanwhile, ICVHM, as a comprehensive indicator, can better identify AR, which proves that better results can be achieved by applying ICVHM to prevent and identify AR.

In subgroup analyses, we observed a trend association between ICVHM scores and AR, the *P* value for the trend was < 0.001 for each subgroup, and we found interactions between ICVHM scores and age, gender, and race. The OR of AR was higher in the age ≤ 65 years old than the elderly, especially in participants with ICVHM score ≥ 5, which indicated that ICVHM was more closely related to AR in the non-elderly population, and it suggested the importance of early prevention of AR. Previous researches had shown that females were more susceptible to the impact of AR [42, 43]. In females, ICVHM scores were more associated with AR than in males, possibly due to differences in genetics and sex hormone secretion affecting the development and activity of the immune system [44], as well as the effect of hormones on structural changes in bones and joints [45]. The finding provided important implications for AR prevention among the specific population of females. Similarly, the association of ICVHM scores with AR was stronger in other race group than in non-Hispanic whites, which may be influenced by social status, health insurance, lifestyle, and other factors [46]. This study provided new ideas for further research on the association between ICVHM scores and AR in different demographic characteristics.

We analyzed the association between ICVHM scores and different types of AR and found that different types of AR were negatively correlated with the score of ICVHM and the trend test was meaningful in each subgroup. As mentioned above, this may be related to the influence of multiple indicators in ICVHM on AR [26–33, 35, 36, 38]. Therefore, our findings suggested that higher ICVHM scores may be valuable for preventing AR and its different types. In addition, among the different ICVHM indicators, blood pressure and total cholesterol, blood pressure and blood glucose, BMI and blood pressure have a strong correlation, and by controlling the more strongly correlated indicators, multiple aspects can be controlled simultaneously, which provides a new idea for the prevention of arthritis in the future.

Strengths of the current study include that NHANES was able to ensure the validity of the data because it used strict quality assurance procedures when conducting surveys and had a large representative sample that could be extrapolated to all adults in the U.S. through weighted analysis. The cardiovascular-related indicators used in this study, such as blood pressure, blood glucose, and total cholesterol, were judged to be ideal by the questionnaire and examination, which had high credibility. In addition, the association and interaction between AR and its different types with ICVHM were studied by subgroup stratified analysis, which provides ideas for further analysis.

Our research also had limitations. First, this study used cross-sectional data for analysis, unable to determine the causal sequence. Second, although a range of covariates was controlled for in our study, there is still a risk of unmeasured confounding and measurement errors that may have biased our results. In addition, we excluded some participants with missing data, which may have resulted in the loss of some information. However, this study still has important guiding significance for the prevention of arthritis.

## Conclusion

In conclusion, higher ICVHM scores were associated with a lower prevalence of AR in U.S. adults, and similar findings were observed for OA, RA, and Other AR. These results supported the idea that AR and CVD may share common risk factors and suggested that improving cardiovascular health may prevent AR and its different types of risks. In addition, there were interactions between age, gender, and race with ICVHM on AR. Our findings guide promoting healthy behaviors and health factors, which have important implications for the prevention of AR and its different types.

### Electronic supplementary material

Below is the link to the electronic supplementary material.

Supplementary Material 1

## Data Availability

The dataset supporting the conclusions of this article is available in the National Health and Nutrition Examination Survey (NHANES), [hyperlink to dataset in https://www.cdc.gov/nchs/nhanes/index.htm].
