# Supplementary material for: Ideal cardiovascular health metrics have better identification of arthritis
Source: BMC Public Health. 2024 Jan 8;24:114. doi: 10.1186/s12889-023-17602-7 (PMC10775435; doi:10.1186/s12889-023-17602-7)
Supplement: Supplementary file 1 — Supplementary Material 1 [file 12889_2023_17602_MOESM1_ESM.docx]

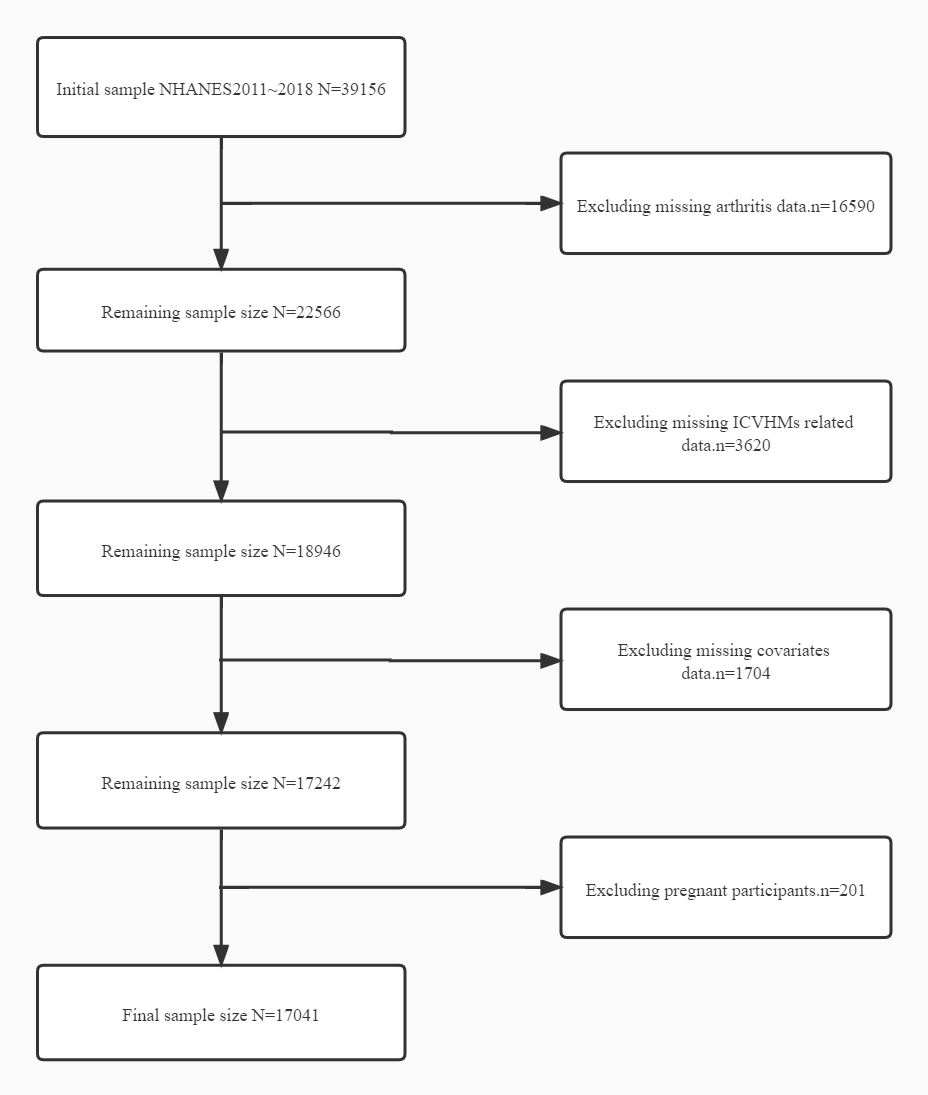


**Figure S1 Data filtering flow chart.**

**Table S1 Component of lifestyle/cardiometabolic factors in the AR.**

| **Lifestyle/ cardiometabolic factors** | **Healthy** | **Unhealthy** |
| --- | --- | --- |
| Smoking | smoking fewer than 100 cigarettes in life | smoking more than 100 cigarettes in life |
|  |  |  |
| Physical activity | In a typical week do moderate or vigorous physical activity | No moderate or vigorous physical activity in a typical week |
|  |  |  |
| Diet | HEI in the top two-fifths of distribution | HEI in the last three-fifths of participants |
|  |  |  |
| BMI | BMI < 25.0 | BMI ≥ 25.0 |
|  |  |  |
| Blood pressure | no hypertension or prehypertension and blood pressure < 120/80 mmHg | hypertension or prehypertension or blood pressure < 120/80 mmHg |
|  |  |  |
| Blood glucose | no hyperglycemia or pre-hyperglycemia and fasting blood glucose < 100 mg/dl | hyperglycemia or pre-hyperglycemia or fasting blood glucose < 100 mg/dl |
|  |  |  |
| Total cholesterol | no high cholesterol level and total cholesterol < 200 mg/dl | high cholesterol level or total cholesterol < 200 mg/dl |


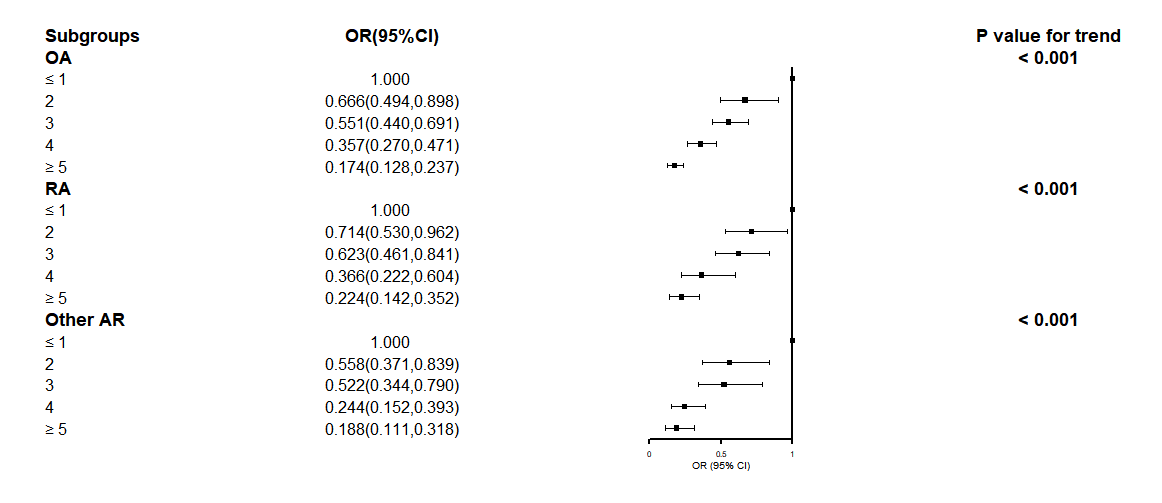


**Figure S2 Relationship between ICVHM scores and different types of AR.**

* Trend test for ICVHMs scores were performed by treating the score of ideal cardiovascular health metrics as a continuous variable.

**
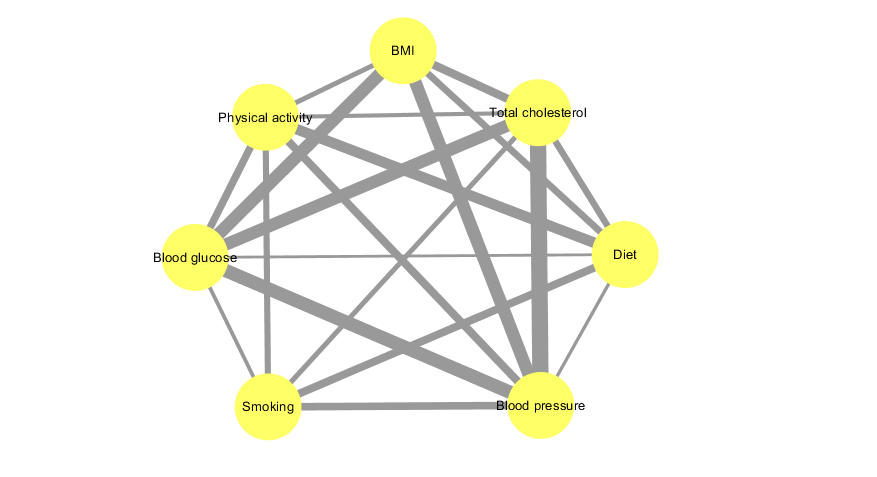
**

**Figure S3 Network of independent variables derived from mixed graphical models.**

**Table S2 Adjacency matrix for pairwise interaction of ICVHM.**

|  | **Smoking** | **Diet** | **Physical activity** | **BMI** | **Blood pressure** | **Total cholesterol** | **Blood glucose** |
| --- | --- | --- | --- | --- | --- | --- | --- |
| **Smoking** | 0.00 |  |  |  |  |  |  |
| **Diet** | 0.18 | 0.00 |  |  |  |  |  |
| **Physical activity** | 0.12 | 0.26 | 0.00 |  |  |  |  |
| **BMI** | 0.00 | 0.14 | 0.09 | 0.00 |  |  |  |
| **Blood pressure** | 0.17 | 0.04 | 0.18 | 0.32 | 0.00 |  |  |
| **Total cholesterol** | 0.09 | 0.14 | 0.06 | 0.19 | 0.43 | 0.00 |  |
| **Blood glucose** | 0.05 | 0.02 | 0.16 | 0.32 | 0.34 | 0.31 | 0.00 |
